# Translating research into policy: lessons learned from eclampsia treatment and malaria control in three southern African countries

**DOI:** 10.1186/1478-4505-7-31

**Published:** 2009-12-30

**Authors:** Godfrey Woelk, Karen Daniels, Julie Cliff, Simon Lewin, Esperança Sevene, Benedita Fernandes, Alda Mariano, Sheillah Matinhure, Andrew D Oxman, John N Lavis, Cecilia Stålsby Lundborg

**Affiliations:** 1 Department of Community Medicine, University of Zimbabwe, PO Box A178 Avondale, Harare, Zimbabwe; 2 RTI International, PO Box 12194, Research Triangle Park, North Carolina 27709-2194, USA; 3 Health Systems Research Unit, Medical Research Council of South Africa, PO Box 19070, Tygerberg, 7505, South Africa; 4 Department of Community Health, Faculty of Medicine, Eduardo Mondlane University, CP 257, Maputo, Mozambique; 5 Norwegian Knowledge Centre for the Health Services, PO Box 7004, St Olavs plass, N-0130 Oslo, Norway; 6 Department of Pharmacology, Faculty of Medicine, Eduardo Mondlane University, CP 257, Maputo, Mozambique; 7 National Institute of Health, Ministry of Health, CP 264, Maputo, Mozambique; 8 Department of Clinical Epidemiology and Biostatistics, McMaster University, 1200 Main Street West, Hamilton, Ontario L8N 3Z5, Canada; 9 Division of Global Health (IHCAR), Karolinska Institutet, Nobels väg 9, SE 171 77 Stockholm, Sweden

## Abstract

**Background:**

Little is known about the process of knowledge translation in low- and middle-income countries. We studied policymaking processes in Mozambique, South Africa and Zimbabwe to understand the factors affecting the use of research evidence in national policy development, with a particular focus on the findings from randomized control trials (RCTs). We examined two cases: the use of magnesium sulphate (MgSO_4_) in the treatment of eclampsia in pregnancy (a clinical case); and the use of insecticide treated bed nets and indoor residual household spraying for malaria vector control (a public health case).

**Methods:**

We used a qualitative case-study methodology to explore the policy making process. We carried out key informants interviews with a range of research and policy stakeholders in each country, reviewed documents and developed timelines of key events. Using an iterative approach, we undertook a thematic analysis of the data.

**Findings:**

Prior experience of particular interventions, local champions, stakeholders and international networks, and the involvement of researchers in policy development were important in knowledge translation for both case studies. Key differences across the two case studies included the nature of the evidence, with clear evidence of efficacy for MgSO_4 _and ongoing debate regarding the efficacy of bed nets compared with spraying; local researcher involvement in international evidence production, which was stronger for MgSO_4 _than for malaria vector control; and a long-standing culture of evidence-based health care within obstetrics. Other differences were the importance of bureaucratic processes for clinical regulatory approval of MgSO_4_, and regional networks and political interests for malaria control. In contrast to treatment policies for eclampsia, a diverse group of stakeholders with varied interests, differing in their use and interpretation of evidence, was involved in malaria policy decisions in the three countries.

**Conclusion:**

Translating research knowledge into policy is a complex and context sensitive process. Researchers aiming to enhance knowledge translation need to be aware of factors influencing the demand for different types of research; interact and work closely with key policy stakeholders, networks and local champions; and acknowledge the roles of important interest groups.

## Background

The evidence-based medicine approach, which focused initially on clinical decision-making, has more recently been extended to policy and management decisions, where it is sometimes referred to as 'evidence-based' or 'evidence-informed' policy making [1-3]. Increasingly, the use of global research evidence is being seen as a key component of policy making processes and it has been suggested widely that health goals are more likely to be achieved by policies that are informed by rigorous research evidence [4-8]. The evidence-informed policy making approach suggests that research evidence from the evaluation of health care interventions, particularly evidence from systematic reviews of randomised controlled trials (RCTs), is the most robust form of evidence for informing policy decisions about the allocation of resources to services or programmes as well as decisions on how to deliver and finance these services [9-11]. However, there is still uncertainty about how research evidence is used by policy makers and how best to ensure that available knowledge is translated into policies and actions.

Knowledge translation - defined as the "exchange, synthesis, and effective communication of reliable and relevant research results" [8] p.140) is particularly pertinent in low- and middle-income countries (LMICs), which face scarce resources as well as high disease burdens. Effective and affordable interventions are available for many of the health problems contributing to the disease burden in these countries. However, this has not led to the automatic translation of research evidence into policy-making [4,12-14]; often these interventions are not implemented or are discarded in favour of unproven interventions.

As three publications from the World Health Organization indicate [8,15,16], growing attention is being paid to knowledge translation in global health. Studies suggest that the relationship between knowledge production (research that generates evidence) and knowledge translation is complex [17,18] with a multitude of factors operating at the individual, organizational, systems and contextual levels [19].

This paper aims to contribute to understanding the process of knowledge translation in LMICs by describing the factors affecting the use of research findings, particularly findings from RCTs, in national policy development. It also explores how actors in the policy process understand the notion of 'evidence' for decision-making. The paper focuses on policy making for two case studies: the use of magnesium sulphate (MgSO_4_) in the treatment of eclampsia and severe pre-eclampsia in pregnancy; and the use of insecticide treated bed nets and indoor residual household spraying for malaria vector control. These case studies represent two different types of interventions: a hospital or clinically based intervention for the treatment of eclampsia and a public health or community based intervention in the case of malaria control. The study explores and compares the perceptions of stakeholders involved in research and policy making in the two cases in Mozambique, South Africa and Zimbabwe.

## The case studies

### MgSO_4 _for the treatment of eclampsia and severe pre-eclampsia

Pre-eclampsia and eclampsia are important contributors to maternal and infant morbidity and mortality in low-income countries [20-22]. Strong evidence is available from RCTs of the effectiveness of MgSO_4 _for women with eclampsia and severe pre-eclampsia [23-27]. However, there is concern that this safe, inexpensive drug may still not be available or used widely in many countries [18,28-30]. Ensuring that policies and actions are informed by these RCT results could benefit many women.

### Insecticide treated bed nets compared with indoor residual spraying for the control of malaria

Malaria remains a major contributor to the burden of disease in low-income countries [31]. RCTs have demonstrated the effectiveness of insecticide treated bed nets in reducing malaria incidence in endemic regions [32]. However, there are still controversies regarding the sustainability of bed net programmes and their relative effectiveness compared to traditional indoor residual household spraying with insecticides [33-35]. Decision-makers need to assess these uncertainties in developing policies and scaling up interventions for malaria prevention.

## Methods

We used a qualitative case-study methodology to explore evidence uptake in the policy making process at national level [36]. We did not consider how policies were implemented in clinical medicine or public health but rather focused on the upstream policy making process. In each country, we undertook key informant interviews with key stakeholders (Table 1), reviewed documents and developed timelines of key events. The regionally-based research team consisted of four researchers in Mozambique, and two each in South Africa and Zimbabwe.

**Table 1:** Profile of study respondents

Country	Number of respondents	
	
	MgSO_4 _case study	Malaria case study
**Mozambique**		
Government health officials	4	4
Pharmaceutical policy makers*	5	
NGOs		3
Clinicians/researchers	5	7
International/bilateral agencies		5

**South Africa:**		
Government health officials	5	8
NGOs		2
Clinicians/researchers	10	4
International/bilateral agencies		1

**Zimbabwe**		
Government health officials	2	4
Pharmaceutical policy makers*	7	
Pharmaceutical company representatives	3	
Clinician/researchers	7	2
International/bilateral agencies		4

**Researchers based outside of the study settings**	1	3

*Pharmaceutical policy makers sat on national bodies that determined policy.

### Country selection

These three countries were partners in PraCTiHC (Pragmatic Randomized Controlled Trials in Health Care), a project funded by the European Commission. They are low- and middle-income countries, where eclampsia and malaria are important health problems and where health care systems and policies have often undergone a significant process of re-evaluation after independence or regime change. Although there are significant regional similarities between the three study countries, there are also differences in health systems and resources; in current and past policies with regard to malaria control and the treatment of preclampsia/eclampsia; and in their relationships to national and international policy networks. Including these countries helped to illuminate the similarities and differences in policy making across different settings, thereby improving the generalisability of the study findings.

### Data Collection

#### Document Review

We reviewed documents that provided insight into policy processes at country level. These included both formal policy documents and other official documents such as treatment guidelines, essential drugs lists, circulars, and minutes of meetings.

#### Key Informant Interviews

We conducted interviews between April 2004 and March 2005, based on an interview guide that explored the policy development process. This guide was translated into Portuguese in Mozambique, and most interviews in this country were conducted in this language. English was the language used for the interviews in South Africa and Zimbabwe. Interviews took about 45 minutes. We audio-recorded each interview and transcribed the recordings in their original language.

We used a combination of purposive and snowball sampling to select key informants [37], based on their involvement in research or policy making for each case study (Table 1). An iterative approach to data collection and analysis was used, with themes and issues emerging from early interviews explored further in later interviews. We stopped recruiting respondents once we felt that we had reached data saturation [37].

#### Timelines

For each policy case study, we constructed a timeline of key events in the policy process, including important meetings and the publication of policy documents and research findings. Initially we drafted each timeline based on available documents and on our knowledge of each policy context. Each timeline was then refined using information gained from the document review and interviews. We further corroborated dates and events by searching the internet and through consultation with colleagues familiar with the events.

### Data Analysis and Interpretation

In order to manage the large data set generated, we used a multilayered approach to data analysis. Analysis began with the data generated from the key informant interviews. Country level researchers read and annotated each transcript and identified preliminary themes. These themes reflected issues arising from the interviews and document analyses. We then held a joint workshop where we discussed broad themes emerging across the country data, thus devising a preliminary coding scheme to guide further thematic analysis. These steps were taken to harmonize the analysis process across the study sites. It also facilitated later cross-country and cross-case study analysis. We then undertook further thematic analysis of the data, based on this coding scheme. Once coded, we grouped data extracts from each transcript together under the appropriate category. After reading and re-reading the coded data, a narrative account of the findings was developed. For the write-up, we selected data extracts illustrative of the key themes. To enhance the validity of the study, our research process included internal training of the research team, respondent validation or member checks and attention to negative cases [38]. In addition, we paid considerable attention to exploring similarities and differences in knowledge translation processes between the two case studies and across the three countries.

### Ethical approval

The study received approval from ethics committees at the Medical Research Council of South Africa, the Medical Research Council of Zimbabwe and the Comité Nacional de Bioética para a Saúde in Mozambique (the study countries); and at the London School of Hygiene and Tropical Medicine and the World Health Organization (one of the research funders). After being provided with information, each respondent gave written informed consent for interview. In reporting, we have removed any potential identifiers.

## Results

We present the findings for each of the case studies - MgSO_4 _and malaria - across the three countries. For the purposes of comparison, we have organised the findings in each section around the key issues for knowledge translation emerging from our data. These findings are also summarised in Table 2.

**Table 2:** Summary of study findings

Factors identified as influencing the use of research in policymaking	Case study 1: MgSO_4 _for eclampsia	Case study 2: Bed nets versus spraying for malaria	**Factors found in systematic reviews to influence the use of research in policymaking **[11,13]	Comments
**Research evidence**	High quality evidence from RCTs that supported the effectiveness of MgSO_4 _as first line treatment.	Head-to-head comparisons in RCTs did not find an important difference in effectiveness.	- Timeliness, perceived relevance and quality of the research - Trust in the research and researchers - Availability of research summaries with clear recommendations - Use of jargon and only publishing for a scientific audience	- The perceived relevance and quality of the research on MgSO_4 _was high, whereas the evidence for bed nets was not perceived to be relevant. More weight was given to local experience than to evidence from RCTs for malaria control. - Availability and timeliness of the research were not identified as important for either case.

**Involvement of local researchers in evidence production**	Leading obstetricians in all three countries were involved in the trials.	Researchers in South Africa involved in one comparative trial. Researchers in Mozambique were involved in bed net research.	This was not identified as an important factor in either of the reviews cited above	Involvement in trials may be an important influence directly or through other routes. These other routes include champions, international networks, prior experience and the promotion of a culture of evidence-based health care.

**Prior clinical or public health experience**	Prior experience in the use of specific drugs for eclampsia may explain, in part, differences in policy between the three countries.	Prior experience heavily influenced support for spraying and inhibited policies favouring the use of bed nets.	Policy confirmatory research	The extent to which the research confirmed or challenged prior experience was important for both cases.

**Research and policy champions**	Obstetrician researchers championed MgSO_4 _in all three countries, but in Zimbabwe the key champion emigrated prior to development of a policy supporting MgSO_4 _as the first line drug.	Researchers regionally, particularly in South Africa, championed spraying, whereas researchers in Mozambique championed bed nets.	Neither review specifically identified the role of champions in promoting the use of research evidence, although both found interactions and trust between policymakers and researchers to be the most commonly identified factor supporting research use.	In both cases senior researchers actively advocated for specific policies. In the malaria case, researchers in South Africa and Zimbabwe advocated spraying rather than bed nets. In Mozambique, they advocated nets.

**International networks, organisations and other stakeholders**	International networks that influenced MgSO4 policy were largely evidence-based, such as the Cochrane Collaboration.	A wide range of stakeholders and international organizations with differing interests, including bilateral donors, (e.g. DFID, JICA), and multilateral agencies, (e.g. WHO, UNICEF and the Roll Back Malaria Partnership), influenced malaria policy in Mozambique and Zimbabwe. This was partially due to donor dependence.	Neither review identified stakeholder interests or international networks as being important factors that affect the use of research, although community pressure was identified as an important factor.	It is likely that international organizations play a more important role in policy development in many LMICs compared to high-income countries, due to donor dependence.

**Regional networks of policy makers and researchers**	Regional networks of policy makers and researchers did not emerge as an important factor.	Regional networks of policy makers and researchers emerged as a key factor influencing malaria policy.	Existence of policy networks	This difference between the two cases may reflect differences between public health policies, particularly for vector-borne diseases that cross borders, and policies for clinical interventions. In addition, powerful champions promoted regional networks for malaria policy and control.

**Involvement of researchers in policy making**	Researchers played an important role in policy development in all three countries.	Researchers played an important role in policy development in all three countries.	Interactions and trust between policymakers and researchers.	There was extensive interaction between researchers and policy makers in both cases, and researchers also moved between the research and policy environments. This level of interchange may be more common in LMICs than in high- income countries.

**Culture of evidence-based health care within specific health domains**	This emerged as an important factor supporting the uptake of research findings for MgSO^4^, due to the strong culture of evidence-based health care in obstetrics.	The culture in relationship to evidence varied for malaria, with greater emphasis on local observational evidence. Differences in malaria epidemiology contributed to this emphasis.	Skill and attitudes of those receiving the research	The greater focus on local conditions and evidence may be more typical for public health and communicable diseases management particularly.

**Political and bureaucratic processes**	Bureaucratic processes can in part explain the failure to include MgSO_4 _in the national formulary in Mozambique even though it was recommended [30].	Political processes at national, regional and international levels may have contributed to the continuation of policies that failed to promote the use of bed nets.	- Bureaucratic process including power and budget struggles and conflicts - Management support	Bureaucratic processes emerged as being potentially important for MgSO_4_, whereas political processes appeared more important for malaria policies.

**Events within the wider political environment**	Although this did not emerge as an important factor, political and economic instability may have influenced policy in Zimbabwe.	Political and economic changes influenced policy in several ways: through South Africa becoming influential in regional politics; through lobbying by interest groups; and with regard to ideological and political perceptions of spraying and bed nets.	The political environment including political stability and community pressure.	External political events can be a limiting factor, but are perhaps more important in public health than in clinical medicine.

### MgSO_4 _in the treatment of eclampsia and severe pre-eclampsia

#### The research evidence

Landmark trials, subsequently incorporated into Cochrane reviews, have demonstrated the efficacy of MgSO_4 _in the treatment of eclampsia and pre-eclampsia in pregnancy [23-27]. South Africa and Zimbabwe participated in these trials, which presented high quality evidence for the use of this drug as a first line therapeutic.

#### Local involvement in evidence production

In all three countries, leading obstetric departments were involved in trials of MgSO_4_, and the obstetricians concerned participated in policy formulation, influencing uptake of these research findings. In particular, senior obstetricians and other researchers in South Africa and Zimbabwe were involved in the Collaborative Eclampsia and Magpie trials [26,27]. This enhanced the credibility of the trial results within these settings:

*"I think it [the Collaborative Eclampsia Trial] was presented at a number of local meetings. ... the leader in South Africa of that trial, ... he presented it at many meetings and so it was a well known research in this country and in Zimbabwe." *(Clinician/researcher, South Africa)

Involvement in these trials allowed local researchers to gain further experience in the use of MgSO_4 _for the treatment of eclampsia and pre-eclampsia. However, respondents reported that adverse outcomes experienced by patients receiving MgSO_4 _in the Collaborative Eclampsia Trial in Zimbabwe raised concerns amongst clinicians regarding its safety. This was thought to have inhibited its uptake as first line treatment for eclampsia in the country.

Respondents in Mozambique reported that a trial conducted locally in 1989 showed MgSO_4 _to be superior to diazepam for the treatment of eclampsia. Although this study was never published, it was considered important in convincing obstetricians to use the drug:

*"We developed the trial up to a certain point, but it was obvious that the patients treated with magnesium sulphate had a lower mortality, and they awoke more rapidly from comas and convulsions ..." *(Clinician/researcher, Mozambique)

Interactions between research - both local and international - and experiential knowledge therefore influenced researcher-clinicians' acceptance of trial findings on the effectiveness of MgSO_4_; the value attributed to them; and the uptake of these findings into policies.

#### Prior practices and beliefs

While research knowledge was important in shaping policy and practice, experiential knowledge also played a key role. Based on their clinical training, obstetricians in South Africa and Mozambique tended to follow the obstetric schools that promoted the use of MgSO_4 _for the management of eclampsia:

*"I think it depended on where people studied. It depended on what people read and it depended on what people believed." *(Clinician/researcher, South Africa)

Because practice prior to the landmark trials was often based on MgSO_4_, many obstetricians noted that they had experienced the clinical success of the drug long before it was shown to be effective in trials:

*"...even in the absence of a randomised study, I think the empirical results were very convincing." *(Clinician/researcher, Mozambique).

Because the evidence from the later Collaborative Eclampsia Trial [26] was congruent with existing practice, its uptake into policy was easier.

In Zimbabwe, on the other hand, obstetricians tended, before MgSO_4 _was demonstrated to be effective, to follow the British tradition of using diazepam for eclampsia management:

*"...the older consultants have been trained in Britain and came with what we were doing in Britain, and practising what we were doing in Britain and this has been passed on to the others in the country...." *(Clinician/researcher, Zimbabwe)

*"... diazepam has been there for as long as I have been there, even pre-independence [before 1980], diazepam has been used all along...." *(Clinician/researcher, Zimbabwe)

#### Champions and lobby groups

The role of, and need for, local champions in placing issues on the policy agenda was highlighted. In all three countries, clinical champions lobbied for MgSO_4 _to be included as the first line treatment for eclampsia:

*"Yes, both [clinicians] in private and in public practice [lobbied], some as groups of researchers and others in their individual capacities...." *(Clinician/researcher, Zimbabwe)

Respondents in South Africa suggested that individual and organizational lobbying drew attention initially to the causes of maternal mortality, helping to place maternal and child health on the policy agenda [39,40]. This helped to ensure that the Department of Health prioritised the development of evidence-based policies to improve maternal health. In Zimbabwe, clinical champions were also important in developing guidelines for the treatment of eclampsia. Their importance was illustrated by the fact that the emigration of the local champion for MgSO_4_, before the full acceptance of the drug was realised, was regarded by some as a cause for slowing the process of getting MgSO_4 _into policy and practice for the management of eclampsia.

Lobbying and championing was not limited to the national level. Groups such as the Cochrane Collaboration influenced how local researchers and policy makers thought about evidence-based practice and policy, as we discuss below.

#### Involvement in national and international research networks

In all three countries, academic obstetricians who were key to local policy development were involved in national, regional and international research networks. These networks were influential in building a culture of research and evidence-based medicine through exposing local clinicians to these ideas as they developed internationally. Directly and indirectly, these networks therefore shaped the translation of evidence into policies. For example, our data indicated that proponents of evidence-based obstetrics internationally, including several linked to the then developing Cochrane Collaboration, participated in conferences in South Africa:

*"We actually invited [researchers]*.... *attached to the Oxford Database [the precursor to the Cochrane Library].... So we were sort of, I think from the word go, when the Oxford Database became available for use, we were part of it, we were aware of it, we were using it, and I think quite a few South Africans became involved on their editorial board and as editors or reviewers, or whatever." *(Clinician/researcher, South Africa)

Leading international researchers also spent sabbatical periods, or developed research units, in the study countries. In Mozambique, international researchers working in the capital facilitated access to the international literature and organised scientific exchanges:

*"...studying abroad, having scientific interchanges not just based on the diseases here, is important. The doctors who come to work here help in training the Mozambican doctors who work with them"*. (Clinician/researcher, Mozambique)

These networks built links to key research taking place internationally and to international policy debates on treatment. Later these networks were also important in recruiting researchers from the study countries into international trials of MgSO_4_.

#### Involvement of researchers in policy making

Key to knowledge translation in the case of MgSO_4 _was the interface between researchers and policy-making. In all three countries, academic obstetricians who were also active as researchers played important roles in policy development. These obstetricians worked with government officials to draft and review policies, often through expert groups. In Mozambique and Zimbabwe, they were often given policy-making responsibilities, as a researcher in Zimbabwe notes:

*"The policy of management of eclampsia is enunciated in the EDLIZ [Essential Drugs List of Zimbabwe] and is a process of consultation within affiliated disciplines to arrive at compilation of the document. Obstetricians and gynaecologists contributed to this through a nominated point person who worked with the Ministry Steering Committee to draw up this document." *(Clinician/researcher, Zimbabwe)

Government officials also gave researchers other tasks, such as drawing up operational plans, training and supervision, which contributed to close working relationships. In Mozambique, some academic obstetricians also occupied key positions in the Ministry of Health.

In contrast, limited opportunities existed for academics to engage with government in South Africa prior to the change of government in 1994. The new government, however, prioritized maternal and child health and employed into its ranks key members of the national academic obstetric community. This created opportunities for academic engagement in this sector with key policy-making committees being chaired by academic obstetricians. A clinician researcher described this change in researcher involvement in policy-making:

*"...I've worked in obstetrics before the new government and after, and ... before there was never the ability to talk about national policies or anything like that. Certainly afterwards [after 1994] there's been a great movement to be able to do that, to participate and to make guidelines." *(Clinician/researcher, South Africa)

In all three countries, obstetricians thus had ready access to policy makers and were part of tight-knit policy communities. The closeness of academic obstetricians in each country to the policy making process suggests that they were potentially key conduits for knowledge translation.

#### A culture of evidence based obstetrics

Attempts have been made internationally to develop a culture of evidence-based research and practice within obstetrics [41]. In the study settings, a culture of basing practice on research findings preceded the availability of evidence from RCTs. We have already described how local researchers became linked into international evidence-based medicine networks. The success of these international initiatives in the study countries was clear from our interviews, with most respondents expressing strong views on the importance of using and generating evidence:

*"...so evidence, that sort of thing was really grasped with both hands. I think a lot of our research is clinical. So trials are our - if you want to do research -is our bread and butter ... I don't think there's any ...O & G [obstetric and gynaecology] academic institution which doesn't use Cochrane extensively." *(Clinician/researcher, South Africa)

Most respondents embraced strongly a culture of evidence-based medicine, believing that policies should be based on RCT findings. Since many of these clinicians were key to the policy making process, their definitions of evidence influenced strongly the sorts of information considered during policy development. Respondents placed high value on evidence from RCTs, including the international collaborative trials [26,27]. Respondents noted that before evidence from these trials became available, other forms of research information such as the Pritchard case series on the treatment of eclampsia [42,43] were relied upon:

*"... in a very famous series of cases of Pritchard, they had 300 consecutive cases of eclampsia without any maternal deaths ...with magnesium sulphate. That's clinical proof..." *(Clinician/researcher, Mozambique)

### Insecticide treated bed nets compared with indoor residual household spraying for malaria vector control

#### The evidence

Indoor residual spraying and insecticide-treated nets have been demonstrated to be effective across a wide range of settings. However, few randomised trials have compared directly the efficacy of bed nets and indoor residual insecticide spraying and their comparative cost-effectiveness depends on the context in which they are implemented. It is therefore difficult to justify one approach over another based on the available evidence [44,45]. At the time of the study, there had also been no trials directly comparing different insecticides. In addition, long-term impregnated nets had not been widely distributed in the study settings.

#### The stakeholders and international agencies

A large and diverse group of stakeholders was involved in decisions on malaria policy in the three countries. Important players included government officials; multilateral agencies (particularly the WHO and UNICEF); partnerships such as the Roll Back Malaria Partnership (which includes WHO, UNICEF, UNDP, the World Bank and a wide range of other NGOs); foundations; donors (such as the UK Department for International Development (DFID); the Japan International Co-operation Agency (JICA); and the Global Fund against AIDS, Tuberculosis and Malaria); academic and private sector institutions; NGOs (such as environmental organisations); political actors; and commercial actors (such as insecticide manufacturers). These stakeholders expressed varied and contested interests, differed in their use and interpretation of evidence, and promoted different malaria control policies, as we describe elsewhere [46]. Contested issues included which insecticide to use (all three countries) and whether to use nets or spraying (Mozambique). Political and commercial interests were also evident, and these interests attempted to influence both policy makers and researchers. For example, the tobacco lobby in Zimbabwe was important in the decision to stop using DDT because of fears that the pesticide would contaminate the tobacco crop.

*" [The] tobacco commercial farming sector lobbied government against use of DDT because the buyers of Zimbabwe tobacco abroad were saying that, if they found traces of DDT in tobacco, they would not buy Zimbabwe tobacco." (*International agency, Zimbabwe)

In Mozambique, younger researchers mostly favoured the introduction of bed nets. This was because they had been drawn into international bed net research networks through their post-graduate studies overseas and through their contacts with international researchers visiting and working in Mozambique. Because of its higher malaria burden, Mozambique was more integrated than were South Africa and Zimbabwe into the international malaria research networks that had undertaken key RCTs on ITNs.

Bilateral donors, multilateral agencies, and international NGOs were important players in shaping malaria control policies, particularly through funding ITN programmes in Mozambique and Zimbabwe:

*"...but of course the insecticide treated nets agenda is also pushed, as you may be aware, very strongly by the bilateral donors, and other players and the UN family and so on. And so the government has accepted their advice as it were, of course to actually use nets ...Nets, to begin with, I think, were an outside sort of influence" *(International agency, Zimbabwe)

*"We can see that nets have become an international fashion... there is a lot of pressure regarding them..." *(Government health official, Mozambique)

Pesticide and net manufacturers also lobbied for their interests and contributed to developing and shaping evidence. For example, pesticide companies had an interest in promoting insecticides other than DDT for spraying, as greater profits were to be made from newer insecticides. These companies therefore sponsored trials in both Mozambique and Zimbabwe [47-49].

A range of NGOs also worked to influence malaria control policies. Environmental lobby groups, both local and international, played important roles in supporting moves away from DDT in South Africa and Zimbabwe in the 1990s and in mobilising evidence for this:

*"The international community, as you probably know, the Greenpeace people and the environmental lobbying groups started putting pressure...we agreed in principle to try reducing the reliance on DDT mainly because of all the things that were published. So those are more or less the reasons why we moved towards reducing DDT." *(Government official, South Africa)

Later, the NGO 'Africa Fighting Malaria' took on the task of lobbying for DDT http://www.fightingmalaria.org. This organization, apparently linked to international neoconservative groups [50], fiercely criticized environmentalists who had pushed for a ban on DDT [51].

#### The role of regional networks of policy makers and researchers

Researchers and policy makers were organised into strong regional networks that were important in sharing ideas and approaches to malaria control. These networks played a key role in maintaining the emphasis on spraying in the region.

Historically, South Africa and Zimbabwe had close links and several key South African researchers and malaria control officers had been trained, and formerly held positions, at the Blair Research Institute in Harare - an important regional malaria research institution. Strong relationships later developed between South African and Mozambican researchers and implementers around the Lubombo Spatial Development Initiative (LSDI), which aimed to improve malaria control in South Africa, Swaziland and neighbouring areas of Mozambique.

#### Involvement of researchers in policy making

Although policy makers may have become aware of research evidence through their own reading, respondents also identified a number of interfaces through which research entered the policy making process. Firstly, researchers were co-opted into formal government advisory committees in all three settings:

*"...certainly we [in the government department] might have the background knowledge into it but we're not currently working in researching malaria all the time... So we felt that we needed to bring and call on expertise from the country to advise us on policy ... and that is the main reason why the decision was taken to put an advisory group together." *(Government official, South Africa)

Consequently, close relationships developed between researchers and health officials responsible for implementing malaria control. These researchers were regarded as experts and evidence uptake was mediated through them.

Secondly, researchers acted in some instances not only as advisors but also as implementers. For example, a senior researcher in South Africa was integrally involved in implementing malaria control initiatives within the LSDI. This initiative, which integrated research and implementation, helped to bring researchers and policy makers together, as one researcher involved in this noted:

*"...we brought service and research together. We thought, 'What's happening in the world? There's a lot of research going on but is it translating into implementation?'. I think that you have to bring those two communities together because the one needs to feed into the other... Over the last ten years, maybe fifteen, we've really worked towards trying to bridge that gap*." (Researcher, Mozambique)

Thirdly, national research bodies had close links with government policy-making bodies in all three countries:

*"I think major advantages that Blair [the Blair Research Institute] has made, or Blair research findings had in influencing policy, is that Blair themselves are part of the national malaria control programme, and Blair do sit in national malaria control programme committees. So the work that they do and their research findings find [their] way almost automatically, naturally into policy and decision making." *(International agency, Zimbabwe)

Knowledge translation for malaria control was facilitated through these different avenues.

#### Defining 'evidence' in the context of malaria control

In contrast to the MgSO_4 _case study, there seemed to be a less visible culture of decision-making for malaria control being based on RCT evidence. Respondents were clear that, for them, what constituted research evidence went beyond RCTs and included experience, surveillance data and expert opinion. Considerable research was undertaken locally in all three countries. This research - broad in its methodological approach - was regarded as important to decision making. For example, the malaria surveillance data collected for many years in Zimbabwe and South Africa were seen as important evidence for shaping policy (see, for example, [52]), particularly in showing the effectiveness of the spraying approaches used in these settings. The weight of this evidence meant that RCTs were not seen as necessary to demonstrate the effectiveness of spraying:

*"But in terms of indoor residual spraying, I think the evidence is not disputable - it's there to see! In the countries where indoor residual spraying is done, the number of malaria deaths are very low, but in countries where indoor residual spraying is not being done, the number of malaria deaths are so high." (*Government health official, Zimbabwe)

In Zimbabwe and South Africa, the international evidence from ITN trials was also seen as distant and not necessarily relevant as it was felt that the local epidemiology of the disease was different to that of the countries in which the trials were conducted:

*"Well I know with nets, even up to now there are a lot of controversies. The epidemiology of malaria in Zimbabwe is quite different from Tanzania, is quite different from the Gambia, or where there is high para-endemicity in those areas" *(International agency, Zimbabwe)

Although a national trial comparing bed nets and spraying was conducted in South Africa in the 1990s [53-55], this was stopped when a malaria epidemic demanded quick action that undermined the trial randomisation.

In addition to concerns about the local applicability of RCT evidence, many policy makers were also clear that ease of implementation and sustainability were key to their decision-making regarding malaria control options. Simply showing success in a RCT was not considered sufficient:

*"Several randomised and controlled studies have already been undertaken which prove effectiveness in reducing mortality, but how can this ideal situation be translated into practical terms, while maintaining effectiveness?... In practical terms and under real conditions,... this is where questions arise. Why are there so many nets distributed, and why are there so many sprayings, and there is no positive impact? ...Research should always be done; otherwise we will not be able to know when an intervention is no longer working." *(Researcher, Mozambique)

*"... after five years of investment in the Gambia (teams, money, very high cost), when they [the researchers] left, it all fell apart. People need to learn the advantage of nets...because the nets give more work. ...it is a long-term thing to create a habit*. (Policy maker, Mozambique)

Some respondents within the malaria control programme also differentiated between research, and surveillance and outbreak investigation:

"*We have very little time for actual research. [...] It's basic problem solving. If there's a small outbreak or a rise in cases in a particular area, you go there and try and evaluate and see what the reason for it is. So you can't study or work in one area for a particular time, which virtually rules out any kind of research." *(Government official, South Africa)

While research evidence was regarded as influential, many respondents saw local experience with varied approaches to malaria control as even more important. Thus, the long history of spraying in the region contributed to a preference for its continued use:

*"Historical evidence - there was plenty...50 years of spraying in South Africa with large areas free from malaria that previously had been malarious areas." *(Researcher, Mozambique)

This contributed to the delayed acceptance of bed nets in Mozambique and Zimbabwe and to scepticism towards this control approach in South Africa.

## Discussion

Our knowledge translation findings are similar to those synthesized by Innvær in a systematic review of research on the use of evidence by health policy makers [13]. Factors identified by this review, such as interaction and trust between policy makers and researchers, are consistent with our findings for MgSO_4_. Similarly, we found the political environment to be important in both the MgSO_4 _and malaria cases: democratization in South Africa was a factor enabling the MgSO_4 _research findings to be taken up into policy. Furthermore, the changed political environment in southern Africa after the democratic elections in South Africa in 1994 affected regional malaria policy. The importance of policy networks involving policy makers and researchers, and the influence of political factors such as local conflicts, were also highlighted as important in a more recent systematic review of factors affecting research utilisation by health policy makers and health care managers [11].

In our study, there were similarities across the case studies regarding the routes through which research entered the policy making process; the influence of lobby groups and champions; and the roles of national, regional and international research and policy networks. In addition, similarities were found in the use of field or clinical experience; conceptions of what constituted "evidence"; and the extent of local involvement in evidence production. However, the relative importance of these factors was different for eclampsia and malaria, as we discuss below.

In both Mozambique and Zimbabwe, and to some extent South Africa, the relatively small number of health care clinicians/researchers, policy makers and institutions enabled a greater degree of interaction between them and contributed to the frequent movement of researchers between the research and policy domains. This contrasts with the situation in many high-income countries, where there is often a greater breadth of health personnel and institutions and where it is therefore likely that a lower proportion of senior researchers are involved directly in policy-making.

In the case of policy for the management of eclampsia, obstetricians and researchers in all three countries tended to belong to the same national clinical and research networks and attended the same meetings and conferences. We would therefore suggest that this tightly knit, exclusive group constituted a "policy community" [56,57]. These national policy communities in Zimbabwe and South Africa were linked closely to the Cochrane Collaboration, which played a particularly important role in ensuring a common conceptualisation of "evidence"; providing resources; and lobbying for and facilitating knowledge translation. The existence of these tightly knit national policy communities, consisting of a small number of clinicians with similar backgrounds and training, also enabled consensus regarding what constituted research evidence. As alluded to above, champions were more important for knowledge translation in the eclampsia case study than in the malaria case. We would suggest that this is because the policy communities for eclampsia management were fairly homogenous and small, being formed mainly of obstetricians. Under these circumstances, champions have considerable influence. Other studies have also suggested that such popular opinion leaders have more influence within tightly knit groups [58-60] and may be particularly important in influencing others to adopt new approaches, technologies and treatments.

In contrast, we identified a much wider array of stakeholders within the malaria control domain. These groups, with divergent backgrounds, opinions and interests, more closely resemble *issue networks *in which relations between actors are looser than in policy communities [56,57]. The actors within these networks were an "unruly mélange" [61] of interest groups and political actors, often contesting existing and new interventions and championing different causes, based on ideological, political and commercial interests. In the debates regarding malaria control, which included which insecticide to use and whether or not to use bed nets or spraying, various groups were aligned with different positions. For example, there were pro- and anti-spraying groups among government officials, researchers and politicians in all three countries, and WHO regional and central offices were often on different sides of the ITN debate.

Eclampsia, as a clinical condition, therefore has a narrow "footprint" in the sense that the range of actors involved is fairly narrow - they are more likely to have close working relationships and to have developed a common understanding of the research "evidence". In contrast, malaria control has a broad "footprint", with a very wide range of actors with differing agendas in both the research and policy areas. This makes development of a common understanding of "evidence" more difficult.

Another important factor in knowledge translation was the extent of local involvement in knowledge production. Where local researchers were involved in international multi-centre studies, such as the Collaborative Eclampsia Trial [26], or undertook national studies, the findings were seen as having greater credibility and applicability within the country context. This can be partly explained by the need for research to be relevant to the local epidemiology of disease and to health service organization. However, familiarity with the intervention was also important and, for example, was one of the reasons why bed nets did not receive significant attention from policy makers within the National Department of Health in South Africa.

The overall political and economic context in which knowledge translation took place was also important. The change of government in 1994 in South Africa, for example, brought in a new cohort of policy makers who questioned the longstanding use of DDT for spraying. This influenced the subsequent decision to switch to the use of pyrethroid insecticides for spraying. Similarly, this change in the political context also resulted in far greater emphasis being placed on maternal and child health. This in turn, provided an opportunity to introduce national guidelines for the management of eclampsia based on MgSO_4_. Such "policy windows" [62] are important in understanding why knowledge translation took place in specific context at particular times.

In exploring the role of research evidence in policy-making, this study raises a number of methodological issues. Firstly, it draws heavily on the accounts of respondents. A strength of this approach is that the data represent the views of those closely involved in formulating the policies studied and therefore provide valuable insights into policy processes. However, we recognise that such accounts are inevitably influenced by respondents' position at the time of the event, their position now, their relationship with the researchers, the shifts that they may have made over time between organizations and their memory of particular events [63]. To address this, we attempted to triangulate interview data with information from other sources. Secondly, the purposive sampling draws in part on identifying respondents through others familiar with the policymaking process, and this may have yielded a sample of people sharing similar opinions. To avoid this, we actively sought respondents with differing views and explored negative cases [38]. Thirdly, this study did not address policy implementation, focusing rather on policy development. Further studies are required to explore the ways in which policies for maternal health and malaria control have been implemented in the field. Fourth, this paper attempts to summarise findings across three countries and two case studies and therefore cannot do full justice to the depth of data collected in each site. More detailed reports of components of this work are available [30,46,64]. Finally, the case study approach, like many qualitative methods, has been criticized for being unable to produce generalisations. We would argue, though, that the approach allows theoretical generalisability [65] and provides insights into the ways in which knowledge informs policy making in 'real life' contexts [66].

Key lessons learnt from the study are:

• There is openness among policy stakeholders to considering research findings. International efforts to support the use of research evidence in LMICs should therefore continue.

• Local researchers were more open to the findings of research in which they had been involved.

• Local champions are important and are a potential route for facilitating knowledge transfer. They should therefore be supported.

• National, regional and international networks appear to be very important in both shaping ideas about what constitutes evidence and in acting as a conduit for transfer of research findings. This can have both positive and negative impacts. For example, views regarding the effectiveness of spraying within policy networks operating in Zimbabwe and South Africa may have reduced openness to considering the use of bed nets.

• Context is an important filter for the translation of knowledge at local levels. Issues such as the local applicability of evidence, and the extent to which proposed policies differ from what is currently believed or the status quo, are aspects of this. Strong international evidence may therefore not always be locally accepted.

• Skills and ability to act on research evidence was present in all of the study settings. However, the capacity for absorption was limited by human and other resource constraints. For example, knowledge translation was often dependant on a few key people or on a particular array of conditions/circumstances. The process is therefore a fragile one.

## Conclusion

The World Health Organization has noted that "Stronger emphasis should be placed on translating knowledge into action to improve public health by bridging the gap between what is known and what is actually being done." [8] p. xv). This study illustrates that translating research knowledge into policy and practice is a complex and context sensitive process. Interaction and trust between policymakers and researchers were important factors in the use of research in policymaking in the study settings, as were political and bureaucratic processes, policy confirmatory research and the relevance and quality of the research. Research champions and international networks were also important, though these factors have not been emphasised in systematic reviews of factors affecting policy makers' use of evidence. Efforts to support knowledge translation in LMICs need to both take account of these factors and incorporate evaluation, so that a wider evidence base for knowledge translation in these settings can be developed.

## Competing interests

The authors declare that they have no competing interests.

## Authors' contributions

GW participated in the conception and design of the study and analysis and drafted the manuscript; SL participated in the conception and design of the study and data collection and analysis, and revised the manuscript; JC participated in the conception and design of the study and in data collection and analysis, and revised the manuscript; KD participated in data collection and analysis, and revised the manuscript; ES participated in data collection and analysis and revised the manuscript; BF participated in data collection and analysis and revised the manuscript; AM participated in data collection and analysis and revised the manuscript; SM participated in data collection and analysis, and revised the manuscript; AO participated in the conception of the study and revised the manuscript; JL assisted with analysis and revised the manuscript; CSL participated in the conception of the study and revised the manuscript. All authors read and approved the final manuscript.
